# Regulating the Crystalline Structure and Ion Affinity of Covalent Organic Frameworks for Enhanced Lithium/Magnesium Separation

**DOI:** 10.3390/biomimetics11030177

**Published:** 2026-03-03

**Authors:** Chuncai Wang, Shiwen Bao, Yanfeng Gong, Lei Yu, Zizhe Xu, Chul. B. Park, Kunyan Sui, Jun Gao, Xueli Liu

**Affiliations:** 1 Key Laboratory of Marine Bio-Based Fibers of Shandong Province, Key Laboratory of Shandong Provincial Universities for Advanced Fibers and Composites, College of Materials Science and Engineering, Qingdao University, Qingdao 266071, China; wangcc@qibebt.ac.cn (C.W.); baosw25@mails.jlu.edu.cn (S.B.); gongyf@qibebt.ac.cn (Y.G.); b24140014@s.upc.edu.cn (L.Y.); xzz771@163.com (Z.X.); park@mie.utoronto.ca (C.B.P.); sky@qdu.edu.cn (K.S.); 2 Qingdao Institute of Bioenergy and Bioprocess Technology, Chinese Academy of Sciences, Qingdao 266101, China; 3 Shandong Energy Institute, Qingdao 266101, China

**Keywords:** membrane separation, ion transport, COF membrane, ion sieving, lithium extraction

## Abstract

Selective ion transport is essential for many applications of membrane separation, such as rare metal and high-value element extraction from complex ionic sources. However, efficient regulation of permeability–selectivity remains a major challenge for advanced ionic transport membranes. Herein, we demonstrate that supercritical CO_2_ (ScCO_2_) drying combined with crown ether functionalization enables precise modulation of crystallinity and ion-specific affinity in covalent organic framework (COF) membranes. The pristine COF membrane prepared by solution casting was amorphous. Owing to its positively charged framework and sub-nanometer pores, the membrane exhibited a high Li^+^ transport rate over Mg^2+^ via a synergistic effect of size exclusion and electrostatic repulsion, resulting in a selectivity of 204. After ScCO_2_ drying, the crystallinity and structural ordering of the COF membrane were significantly enhanced, leading to a 1.5-fold increase in Li^+^ flux, accompanied by a moderate decrease in selectivity to 147. To compensate for this trade-off, 12-crown-4 (12C4) was introduced as a Li^+^ recognition agent into the ScCO_2_-treated membrane, restoring Li^+^/Mg^2+^ selectivity to 187 without compromising Li^+^ flux. Importantly, the selective Li^+^ transport performance was maintained in real salt lake brines. This structural–chemical co-regulation strategy provides a versatile approach for optimizing ion transport membranes in complex separation applications.

## 1. Introduction

Selective ion transport is essential for membrane-based separations and underpins a wide range of applications, including the extraction of high-value metals, energy conversion, and water treatment [1,2,3,4,5,6,7]. Among these, the extraction of lithium from lithium-containing water sources such as salt lakes and seawater via separation membranes—commonly referred to as direct lithium extraction (DLE)—has attracted considerable attention in recent years [8,9,10,11,12,13,14,15]. However, traditional membranes used for ion separation usually lack high selectivity [9,11,16,17,18], making it challenging to obtain high-purity target elements.

Biological ion channels, such as potassium and proton channels, enable the rapid and highly selective transport of target ions [19,20,21], which is fundamental to diverse physiological processes. However, the sensitivity of these channels often results in a loss of bioactivity in vitro, limiting their practical use. Inspired by these systems, artificial ion channels and biomimetic membranes have been extensively explored over the past decades [22,23,24]. By emulating the structural confinement and transport mechanisms of biological channels, such systems can significantly enhance ion selectivity compared with traditional membranes. However, most artificial ion-channel-based membranes still face the intrinsic permeability–selectivity trade-off [4,16]. Although nanoscale or even sub-nanometer confinement combined with surface functionalization can improve selectivity, this approach frequently leads to severely reduced ion flux. Therefore, achieving simultaneous enhancement of flux and selectivity remains a central challenge in artificial ion transport membranes.

Among the new-emerging ion transport materials, covalent organic framework (COF)-based membrane materials show great potential due to their nanoscale pore size and tunable chemical properties [25,26,27,28]. Importantly, the structural arrangement of COF layers plays a critical role in determining ion transport behavior. For instance, Bao et al. reported a COF membrane composed of randomly oriented nanosheets, generating ultra-small effective pores and enabling nearly perfect Li^+^ selectivity [29]. Other studies also suggest that amorphous COF structures offer advantages over crystalline structures in achieving high-selectivity ion sieving, as they lead to a denser membrane with a narrower pore size distribution that significantly enhances the size confinement effect on ions with similar sizes [29]. However, excessively small or tortuous transport pathways typically result in sluggish ion transport, limiting practical applicability.

These studies highlight the importance of COF layer ordering in selective ion transport. In typical COF membrane fabrication, COF nanosheets are first synthesized and subsequently assembled into layered membranes via methods such as vacuum filtration, solution casting, or blade coating [30,31,32,33]. Alternatively, membranes can be formed directly from monomers through casting or interfacial polymerization [34,35,36], though which processes still involve the generation and assembly of COF layers or nanosheets. Therefore, elucidating the relationship between COF layer arrangement and ion transport properties is essential for the rational design of high-performance COF membranes. In biological ion channels, straight and well-defined transport pathways ensure high flux, while molecular recognition groups confer high selectivity. Analogously, improving the crystallinity and ordering of COFs is expected to facilitate ion transport by generating more aligned channels. However, increased crystallinity may increase effective pore sizes or reduce confinement, thereby compromising selectivity. To address this trade-off, functionalizing COFs with Li^+^-specific recognition units, such as crown ethers, may introduce an additional molecular-recognition mechanism.

In this work, we demonstrate regulation of the crystalline structure and ion affinity of COFs for enhanced Li^+^/Mg^2+^ separation. Targeting the industrially important Li^+^/Mg^2+^ separation in salt-lake brines, we employ a positively charged COF to achieve Li^+^ selectivity through synergistic electrostatic repulsion and size exclusion. A simple physical post-treatment—supercritical CO_2_ (ScCO_2_) drying—is introduced to regulate the stacking order and crystallinity of the COF layers, enabling systematic investigation of the relationship between structural ordering, flux, and selectivity. Furthermore, incorporation of Li^+^-selective crown ether moieties restores selectivity while maintaining enhanced flux. This integrated strategy provides new insights into balancing permeability and selectivity in COF-based ion transport membranes.

## 2. Materials and Methods

1,3,5-Triformylphloroglucinol (Tp, 98%), ethidium bromide (EB, 96%) and 12-CROWN 4-ETHER (12C4, 98%) were purchased from Aladdin Co., Ltd., Shanghai, China. Dimethyl sulfoxide (DMSO, 99%), acetone, ethanol, LiCl and MgCl_2_ were supplied by Sinopharm Chemical Reagent Co., Ltd. Dichloromethane (DCM, 99.5%) and dimethylformamide (DMF, 98%) were bought from Meryer Co., Ltd., Shanghai, China. All chemicals were bought with high purity and used as received without further purification.

### 2.1. Fabrication of the TpEB COF Membrane

The TpEB COF nanosheets were synthesized via a one-step solution-phase method. EB (29.61 mg) and Tp (10.51 mg) were separately dissolved in DMSO (3 mL) and then mixed thoroughly to form a homogeneous solution. The reaction mixture was kept undisturbed at room temperature for 24 h to obtain a COF nanosheet colloidal suspension. Subsequently, 6 mL of the COF suspension was cast onto a glass substrate (10 × 10 cm^2^) that had been pre-cleaned by sequential ultrasonication in acetone, ethanol, and deionized (DI) water. The coated substrate was maintained at 60 °C for 3 days to ensure complete solvent evaporation and membrane formation. After immersion in DMSO, the membrane was gently peeled off from the substrate and further soaked in DMSO for 3 h to remove residual unreacted monomers. The membrane was then sequentially rinsed with DMSO, acetone, and DI water, followed by drying at 60 °C. The pristine TpEB COF membrane (approximately 4 μm in thickness) was thus obtained and stored for further use. The membrane thickness was controlled by proportionally increasing the monomer amounts in the reaction solution. Specifically, EB (51.81 mg) and Tp (18.39 mg) were used to obtain a membrane thickness of approximately 7 μm, while EB (81.41 mg) and Tp (28.91 mg) yielded membranes with a thickness of approximately 11 μm.

### 2.2. Fabrication of the ScCO_2–_TpEB COF Membrane

The pristine TpEB COF membrane was subjected to supercritical carbon dioxide (ScCO_2_) drying using a Shianjia (Beijing, China) SCD-380A critical point dryer equipped with high-purity CO_2_ (99.8%). Prior to ScCO_2_ treatment, the membrane was immersed in ethanol for 3 days to replace residual pore solvents. The ethanol-exchanged membrane was then placed in the drying chamber, which was cooled to 2 °C and filled with ethanol. Subsequently, liquid CO_2_ was introduced to replace ethanol in the chamber. The temperature was gradually increased to 40 °C to reach supercritical conditions. During the treatment, the chamber was flushed with fresh CO_2_ four times overnight to ensure complete solvent exchange. Finally, the chamber was slowly depressurized to ambient pressure, yielding the ScCO_2_-treated TpEB COF membrane.

### 2.3. Characterizations

AFM images of the COF nanosheets were obtained using the atomic force microscopy (Bruker Dimension Fastscan, Karlsruhe, Germany) in tapping mode. The lattice structure of the nanosheets was analyzed using a transmission electron microscope (TEM, JEM-F200, JEOL Ltd., Tokyo, Japan). SEM characterizations of the membranes were carried out using a field-emission scanning electron microscope (Hitachi S-4800, Tokyo, Japan), while the element mapping was recorded by energy-dispersive X-ray spectroscopy (EDS) integrated with the SEM. XRD patterns were recorded by an X-ray diffraction diffractometer (Bruker D8 Advance, Karlsruhe, Germany). The chemical compositions of the COF membrane were characterized by Fourier transform infrared spectroscopy (FT-IR, Nicolet 6700, Thermo Scientific, Madison, WI, USA). The contact angle was measured using a contact angle goniometer (Biolin Scientific, Gothenburg, Sweden, Theta Lite).

### 2.4. Ion Selectivity Measurement

The COF membrane was mounted between two polytetrafluoroethylene (PTFE) reservoirs to assemble an osmotic diffusion cell. A feed solution (10 mL) containing LiCl, MgCl_2_, or their mixtures (0.1 M for each cation unless otherwise specified) was added to one reservoir, while 10 mL of deionized (DI) water was placed in the other reservoir as the receiving phase. The concentrations of cations in the permeate solution were determined as a function of time using inductively coupled plasma mass spectrometry (ICP–MS). The ion permeation rate (J) was calculated according to:

$$J=\frac{V}{S}*\frac{dc}{dt}$$

where $\frac{dc}{dt}$ represents the rate of change in ion concentration in the permeate with respect to time, V is the volume of the permeate solution, and S is the effective membrane area (7.065 mm^2^).

The selectivity of one ion species (*i*) over the other ion type (j) was calculated:

$$S_{ij}=\frac{J_{i}}{J_{j}}$$

## 3. Results

### 3.1. Preparation of the ScCO_2–_TpEB COF Membranes

**Figure 1:**
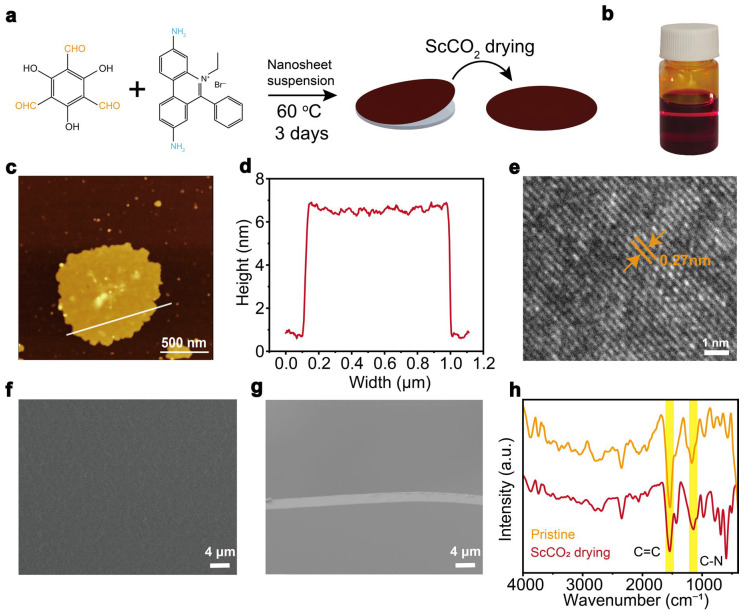
Fabrication of the ScCO_2_-treated COF membrane. (**a**) Schematic illustration of the preparation of ScCO_2_–COF membrane. The monomers were dissolved in DMSO and stood for 3 days to obtain the COF nanosheet dispersion, which was then cast onto a glass substrate to form the pristine COF membrane after drying. After ethanol soaking and ScCO_2_ drying, the ScCO_2_–COF membrane was finally obtained. (**b**) Optical image of the COF nanosheet dispersion. (**c**,**d**) AFM image of the TpEB COF nanosheets (**c**), which were about 6 nm thick with a lateral size of about 1 µm (**d**). (**e**) HR-TEM image of the COF nanosheet, indicating a good crystallization with a d-spacing of 0.27 nm. (**f**,**g**) Surface and cross-sectional SEM images of the ScCO_2_–COF membrane. (**h**) FT-IR spectra of the pristine TpEB COF membrane (yellow curve) and the ScCO_2_–COF membrane (red curve). The lines in Figure 1c represent the size information d of the nanosheets at that location. In Figure 1e, the arrows indicate the d-spacing of the nanosheets.

The pristine TpEB COF membrane was fabricated via a solution-phase synthesis of COF nanosheets, followed by solution casting of the nanosheet dispersion [37]. The as-prepared membrane was subsequently subjected to supercritical carbon dioxide (ScCO_2_) drying to obtain the ScCO_2–_TpEB COF membrane (schematic illustration in Figure 1a; see Section 2.1 for details). The TpEB COF was synthesized by two monomers, namely 1,3,5-triaminoxybenzidine (Tp) and ethidium bromide (EB). EB is a cationic monomer, coupled with Br counter anions, resulting in a positively charged COF. Figure 1b shows the suspension of TpEB COF nanosheets, exhibiting the Tyndall effect and indicating the good dispersion of the nanosheets in DMSO solvent. AFM analysis reveals that the COF nanosheets possess an average lateral size of approximately 1 μm and a thickness of about 6 nm (Figure 1c,d). High-resolution TEM images show that the nanosheets contain both ordered and amorphous regions, primarily located in the central and edge areas, respectively (Figure 1e and Figure S1). Furthermore, TEM elemental mapping [38] confirms the elemental distribution and chemical composition of the COF nanosheets (Figure S2).

After solution casting and vacuum drying, the COF nanosheets assembled into a continuous membrane (Figure S3), where the dynamic covalent bond of imine endows the healing of the frameworks between nanosheets. The pristine TpEB COF membrane was then treated by ScCO_2_ drying after soaking in ethanol for 3 days. Surface SEM images indicate no noticeable morphological changes before and after ScCO_2_ treatment (Figure 1f and Figure S4). The membranes are all defect-free and uniform, with a thickness of about 4 µm (Figure 1g). EDS analysis confirms that the chemical compositions of the COF membranes were nearly the same before and after the ScCO_2_ treatment (Figure S5). In addition, FT-IR analysis [39] of the COF membranes also shows that the COF structures were well synthesized with no obvious difference caused by the drying process (Figure 1h). Both membranes exhibit hydrophilic surface properties (Figure S6), which are favorable for ionic transport.

Moreover, the membrane thickness can be adjusted by changing the monomer concentration proportionally during the synthesis of COF nanosheets (Figure S7). Overall, the fabrication process is straightforward and scalable, enabling large-area membrane preparation depending on the size of the casting substrate.

### 3.2. Selective Li^+^ Transport of the ScCO_2–_TpEB COF Membranes

**Figure 2:**
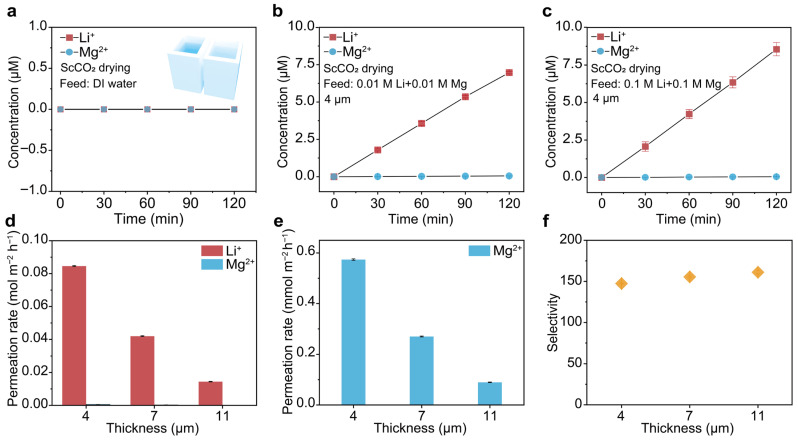
Li^+^/Mg^2+^ selective transport by the ScCO_2_–COF membrane. (**a**) Control test of the ion permeation process. The inset shows the setup for ion permeation tests. The ScCO_2_–COF membrane was mounted between the two reservoirs filled with DI water during the control test. No Li^+^ or Mg^2+^ were detected, ruling out the concern about ionic pollution. (**b**,**c**) Concentrations of Li^+^ and Mg^2+^ in the permeate as a function of permeation time. The feed solution contains 0.01 M LiCl + 0.01 M MgCl_2_ in (**b**), and 0.1 M LiCl + 0.1 M MgCl_2_ in (**c**). The membrane thickness was 4 μm. (**d**) The influence of membrane thickness on the permeation rates of Li^+^ and Mg^2+^ when a 0.1 M equimolar solution was used as the feed. (**e**) The enlarged diagram shows the permeation rates of Mg^2+^ corresponding to those in (**d**). (**f**) The corresponding Li^+^/Mg^2+^ selectivity is concluded from (**d**,**e**). The results show that the ScCO_2_–COF membrane transports Li^+^ faster than Mg^2+^, with considerable Li^+^/Mg^2+^ selectivity around 147. As the membrane thickness increased, the permeation rates of both ions dropped while the selectivity remained relatively stable.

The ion transport experiments were carried out via concentration-gradient-driven diffusion dialysis using a customized H-type device (schematic illustration in the inset of Figure 2a; see Section 2.1 for details). The membrane was mounted between the two reservoirs, while one reservoir was filled with electrolyte solution containing ions and the other reservoir with deionized (DI) water. As this study focused on Li sieving from salt lake water, feed solutions containing Li^+^ and Mg^2+^ cations in their chloride form were used for the permeation.

A control experiment was first performed to exclude potential contamination from the investigated ions by filling both reservoirs with DI water while mounting the COF membrane in between. As shown in Figure 2a, there was indeed no Li^+^ or Mg^2+^ detected during the 2 h of permeation. Subsequently, an equimolar Li^+^/Mg^2+^ solution was used as the feed. As shown in Figure 2b,c, the concentrations of Li^+^ and Mg^2+^ in the permeate increase steadily with time, consistent with the Fickian diffusion law driven by concentration gradient. The results show that the ScCO_2–_TpEB COF membranes transport Li^+^ faster than Mg^2+^. The concentration of Li^+^ reached 4.5 μmol L^−1^ after 2 h of permeation, 133 times higher than that of Mg^2+^, when the feed concentration was 0.01 M (Figure 2b). The resulting Li^+^/Mg^2+^ selectivity was around 133. Upon increasing the feed concentration to 0.1 M, the permeation rate of Li^+^ was also increased (Figure 2c), while the Li^+^/Mg^2+^ selectivity was relatively maintained (Figure S8). The preferential transport of Li^+^ over Mg^2+^ can be attributed to the synergistic effects of charge selectivity and size confinement. The positively charged TpEB COFs generate stronger electrostatic repulsion toward Mg^2+^ than Li^+^, while the sub-nanometer pores impose greater steric hindrance on the larger Mg^2+^ ions, collectively leading to the high Li^+^/Mg^2+^ selectivity of the ScCO_2–_TpEB COF membrane.

The above used ScCO_2–_TpEB COF membrane had a thickness of 4 µm. The influence of membrane thickness on the selectivity of Li^+^/Mg^2+^ transport was further investigated by adjusting the thickness. As shown in Figure 2d,e and Figure S9, the permeate rates of both Li^+^ and Mg^2+^ increased along with the reduction in thickness, which is reasonable as the thinner the membrane, the lower the transfer resistance will be. Meanwhile, the Li^+^/Mg^2+^ selectivity remained around 150 (Figure 2f). Notably, when the membrane thickness was reduced to below 4 μm, the membrane became inhomogeneous and mechanically fragile. Therefore, although the measured selectivity was higher than that of other thicknesses, it is not stable enough for the tests. As a result, 4 µm thick membranes were used for further investigations. We anticipate that by using other preparation methods, such as blading casting, instead of drop casting used here, a thinner membrane with better continuity and stability could be prepared to further improve the Li^+^ flux without compromising the selectivity.

### 3.3. Influence of ScCO_2_ Drying on the Selective Li^+^/Mg^2+^ Transport and the Mechanism Behind

**Figure 3:**
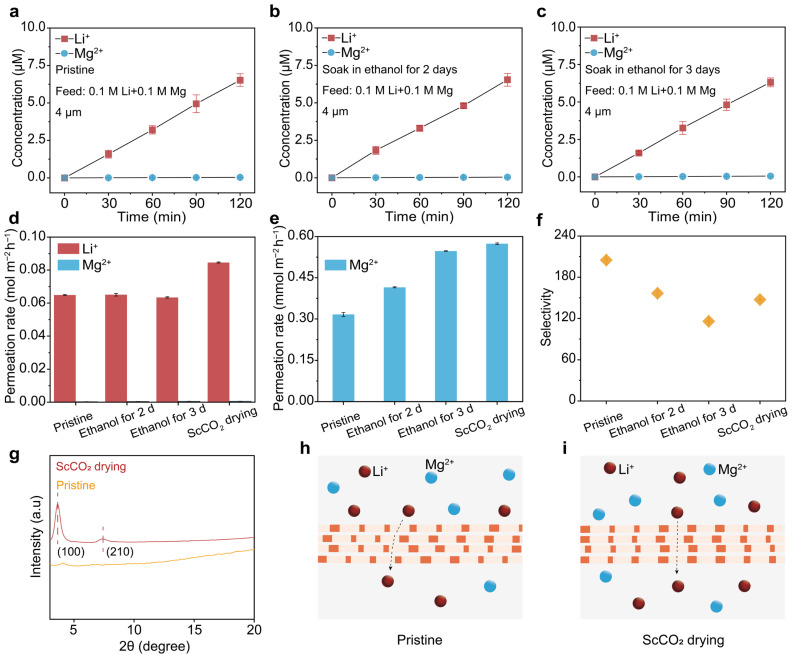
The influence of ScCO_2_ drying on the selective Li^+^/Mg^2+^ transport. (**a**–**c**) Concentrations of Li^+^ and Mg^2+^ in the permeate over time for the pristine TpEB COF membrane (**a**), and the membranes soaked in ethanol for 2 (**b**) and 3 (**c**) days before ScCO_2_ drying. (**d**) Comparison of the permeation rates of Li^+^ and Mg^2+^, for the pristine, ethanol-soaked, and ScCO_2_-treated COF membranes. (**e**) The enlarged diagram shows the permeation rates of Mg^2+^ corresponding to those in (**d**). (**f**) The corresponding Li^+^/Mg^2+^ selectivity is concluded from (**d**,**e**). After ScCO_2_ drying, the membrane showed increased permeation rates for both ions, with a decrease in Li^+^/Mg^2+^ selectivity from about 200 to 150. (**g**) XRD patterns of the pristine TpEB COF membrane (yellow curve) and the ScCO_2_-treated COF membrane (red curve), indicating that the crystallinity of the COF membrane was improved after the ScCO_2_ drying. (**h**,**i**) Schematic illustration of Li^+^/Mg^2+^ transport behavior in pristine TpEB COF membranes and in membranes subjected to ethanol soaking followed by ScCO_2_ drying. The schematic provides a qualitative comparison illustrating the influence of the combined treatment on ion transport, which is consistent with the experimentally observed enhancement in crystallinity and ion flux. The combined treatment leads to a cleaner pore environment and improved structural ordering, facilitating faster Li^+^ transport, while largely preserving ion-sieving selectivity.

To comprehensively evaluate the effect of ScCO_2_ drying on the ion transport properties of TpEB COF membranes, we carried out a series of ion permeation tests of the pristine, ethanol-treated, and ScCO_2_-dried membranes. Figure 3a–c shows the permeated concentrations of Li^+^ and Mg^2+^ as a function of time for the pristine (a) and ethanol-soaked (b–c) membranes, while the permeation results of the ScCO_2_-treated membrane are shown in Figure 2c. Figure 3d,e summarized the corresponding permeation rates of Li^+^ and Mg^2+^ (d) and Mg^2+^ in specific (e). The results show that, along with ethanol soaking, the permeation rates of Li^+^ remained nearly unchanged while the permeation rates of Mg^2+^ were increased gradually, resulting in a decrease in Li^+^/Mg^2+^ selectivity from about 210 to 110 when comparing to the pristine membrane (Figure 3f). After the ScCO_2_ drying, the permeation rates of both Li^+^ and Mg^2+^ were both further enhanced, while the enhancement of Li^+^ flux was more pronounced, resulting in an increase in selectivity from about 110 to 150. Similar trends were observed for membranes with 7 µm thickness (Figures S10 and S11).

The above results indicate that the ScCO_2_ drying procedure can promote ion flux while only causing a marginal change in ion selectivity, a trend commonly observed in ionic and molecular separation membranes and often referred to as a permeability–selectivity trade-off. As the chemical composition was nearly the same for the COF membranes before and after the ScCO_2_ drying procedure (Figure 1g), we attribute the variation in ion transport properties to the microstructural changes in the membrane. For verification, we compared the nanoscale structures of the pristine and ScCO_2–_TpEB COF membranes by XRD characterizations. As shown in Figure 3g, the XRD patterns clearly show that the crystallinity of the COF membrane was significantly enhanced after the ScCO_2_ treatment. The sharper and more intense diffraction peaks of the ScCO_2–_TpEB COF membrane at (100) and (210) crystal planes strongly indicate a more ordered and highly crystalline COF structure, while the pristine membrane was largely amorphous with a very low crystalline degree. Owing to the enhanced crystallinity by ScCO_2_ drying, the overall structural ordering of the COF is improved, which facilitates ion transport and leads to an increased ion flux. (schematic illustrated in Figure 3h,i).

We propose that this crystallinity enhancement can be rationalized by the combined effects of ethanol soaking and subsequent ScCO_2_ drying. Ethanol soaking exchanges and removes strongly polar, solvent-mediated species (e.g., DMSO and loosely bound EB/Br^−^ pairs) from the interlayer and pore regions, thereby weakening solvent-mediated interlayer interactions and facilitating subsequent ordering during ScCO_2_ drying. As shown in Figure S12, the FT-IR spectra indicate that the S=O stretching vibration at 1025 cm^−1^ is significantly attenuated after ethanol soaking treatment. XPS analysis of the Br 3d spectra (Figure S13g) shows a marked decrease in the Br/C ratio, from 0.3 for the pristine membrane to 0.1 after soaking, indicating a reduction in loosely bound Br^−^ species. These results support the role of ethanol soaking in cleaning the interlayer and pore regions. The TGA results in Figure S14 clearly demonstrate that ScCO_2_ drying markedly reduces the weight loss in the low-temperature region (120–250 °C), which is likely attributable to the effective removal of residual DMSO and unreacted monomers trapped within the COF membrane. Under supercritical conditions, CO_2_ exhibits low viscosity and high diffusivity, enabling efficient penetration into COF pores and interlayer regions to extract residual small molecules. Collectively, these effects lead to a cleaner pore environment and improved structural ordering, as reflected by the enhanced crystallinity observed in XRD patterns. As a result, the Li^+^ permeation rate was enhanced after ScCO_2_ treatment, suggesting that ScCO_2_ drying provides an effective strategy to enhance membrane flux without severely compromising ion-sieving selectivity.

### 3.4. Crown Ether Enhanced Li^+^ Selectivity Towards Lithium Extraction from Salt Lakes

**Figure 4:**
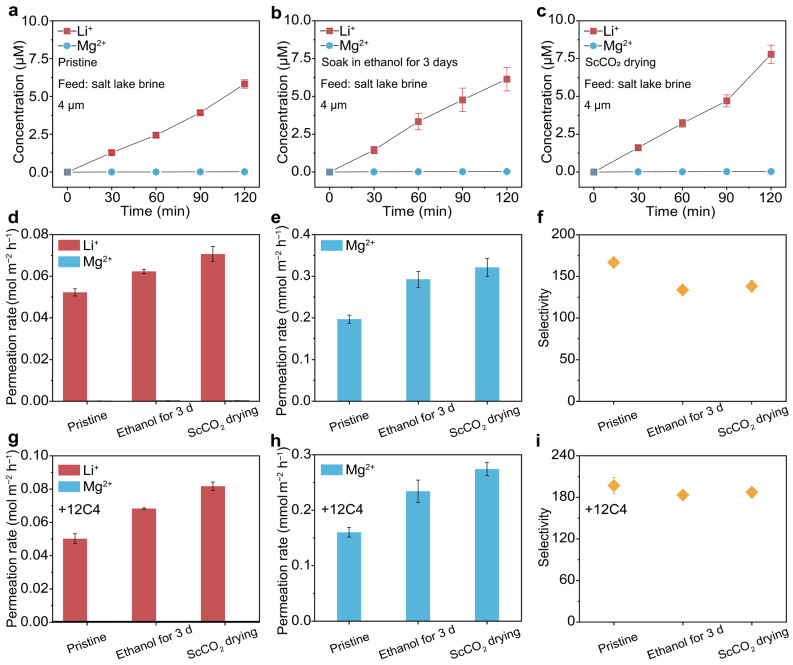
Crown ether recognition enhanced Li^+^/Mg^2+^ separation from salt lake brine. (**a**–**c**) Concentrations of Li^+^ and Mg^2+^ in the permeate as a function of time when brine from Qinghai Salt Lake was used as the feed, for the pristine (**a**), the ethanol-soaked (**b**), and the ScCO_2_-dried (**c**) COF membranes. (**d**) Comparison of the permeation rates of Li^+^ and Mg^2+^ for the pristine, ethanol-soaked, and ScCO_2_ dried COF membranes. (**e**) The enlarged diagram shows the permeation rates of Mg^2+^ corresponding to those in (**d**). (**f**) The corresponding Li^+^/Mg^2+^ selectivity is concluded from (**a**–**e**). The results further confirmed the influence of ScCO_2_ drying on selective Li^+^/Mg^2+^ transport. Moreover, the Li^+^/Mg^2+^ separation capability was maintained when salt lake brine was used as the feed. (**g**) Comparison of the permeation rates of Li^+^ and Mg^2+^, for the pristine, ethanol-soaked, and ScCO_2_-treated COF membranes doped with 12C4 crown ether. (**h**) The enlarged diagram, showing the permeation rates of Mg^2+^ corresponding to those in (**g**). (**i**) The corresponding Li^+^/Mg^2+^ selectivity concluded from (**g**,**h**). The results show that doping with crown ether can enhance the Li^+^/Mg^2+^ selectivity while maintaining the Li^+^ flux, indicating an effective way to further improve the selective Li^+^/Mg^2+^ transport of the ScCO_2_–COF membrane.

To evaluate the selective ion transport performance of the ScCO_2–_TpEB COF membrane under practical conditions, we carried out experiments focusing on the separation of Li^+^ from the saltwater of Qinghai Lake. The composition of Qinghai Lake’s saltwater is detailed in Table S1. The tests were performed the same as before, with one reservoir filled with 10 mL of salt lake brine and the other one filled with DI water. Figure 4a–c presents the concentrations of Li^+^ and Mg^2+^ as a function of time for the pristine (a), the ethanol-soaked (b), and the ScCO_2_-dried (c) COF membranes, while Figure 4d–f summarizes the corresponding permeation rates and selectivity. The results show that the permeate rate of Li^+^ was about 0.05–0.07 mol m^−2^ h^−1^, lower than that of the equimolar model solution utilized above, which is reasonable as the concentration of Li^+^ is much lower in real salt lake water. In contrast, the Li/Mg selectivity was nearly unchanged, with a selectivity of about 138. These results demonstrate the practical feasibility of the ScCO_2–_TpEB COF membrane toward practical Li extraction.

As selectivity is a crucial parameter for membrane applications in Li extraction, we further explored strategies to further improve the Li^+^/Mg^2+^ selectivity of the ScCO_2–_TpEB COF membrane. To this end, a functional molecule with Li recognizing capability, i.e., the 12C4 crown ether, was incorporated into the membrane during the solution casting process after the synthesis of COF nanosheets. For a demonstration, 10 wt.% of crown ether was added to the COF nanosheet suspension for membrane fabrication. The resulting crown ether–doped ScCO2–TpEB COF membrane was then examined for Li^+^ separation from the saltwater of Qinghai Lake. The results are shown in Figure 4g–i and Figure S15. After the incorporation of crown ether, the overall ion permeation flux decreases. This decrease is likely due to a reduction in pore size resulting from the incorporation process. The strong affinity of crown ether for Li^+^ leads to a smaller reduction in Li^+^ flux, while the Mg^2+^ flux is significantly reduced (comparing Figure 4d,e with Figure 4g,h). Consequently, crown ether incorporation preserves the Li^+^ permeation flux while substantially enhancing selectivity. The selectivity increases from 138 of the undoped membrane to 187 (comparing Figure 4f with Figure 4i), which is considerably higher than those of previously reported membranes (Table S2) [16,24,25,33,40,41,42,43], indicating its promising potential for Li^+^ extraction applications.

We further carried out a 1-week continuous separation test to assess the long-term operational stability of the membrane. A Li^+^/Mg^2+^ binary solution whose concentrations are identical to those in the real salt lake brine was used as the feed. Meanwhile, we also used the real salt lake brine as the feed solution for the test, to further reveal the influence of coexisting ions on Li^+^ separation. The results confirmed that the membrane performance remained stable during the long-term separation, with barely changed Li^+^ permeation rates and selectivity ratio for both feeds (Figures S16 and S17). On the other hand, compared to the results of the Li^+^/Mg^2+^ binary feed solution, a slight decrease in Li^+^ permeation rate was observed, while the Li^+^/Mg^2+^ selectivity remained the same when real brine was used as the feed. These results suggest that the coexisting ions (e.g., Na^+^, K^+^, and Ca^2+^) will slightly reduce the Li^+^ flux by competitive transport. Nevertheless, the overall separation performance, especially the Li^+^/Mg^2+^ selectivity, is not markedly compromised.

## 4. Discussion

This work develops a facile strategy to regulate the crystalline structure and ion affinity of COF membranes for efficient Li^+^/Mg^2+^ separation. The pristine TpEB COF membrane, prepared via solution casting, exhibits a Li^+^/Mg^2+^ selectivity of 204 relying on size exclusion and electrostatic repulsion. Supercritical CO_2_ (ScCO_2_) drying significantly enhances the membrane’s crystallinity and interlayer orderliness, boosting Li^+^ flux by 1.5-fold, accompanied by a moderate decrease in selectivity to 147. To synergistically optimize flux and selectivity, doping 12C4 crown ether (a Li^+^-specific recognition agent) into the ScCO_2_-treated membrane elevates the Li^+^/Mg^2+^ selectivity to 187 without compromising Li^+^ permeation. Importantly, the membrane retains excellent separation performance in real salt lake brines, demonstrating its potential for practical lithium extraction for direct lithium extraction. This strategy of combining physical post-treatment (ScCO_2_ drying) and chemical functionalization (crown ether doping) provides a versatile approach to provides a viable strategy to mitigate the flux–selectivity trade-off in ion transport membranes, offering valuable insights for the design of advanced separation materials.

## Data Availability

The data that support the findings of this work are available from the corresponding author upon reasonable request.

## Supplementary Materials

The following supporting information can be downloaded at: https://www.mdpi.com/article/10.3390/biomimetics11030177/s1. Supplementary information regarding the characterization of COF nanosheets and membranes, such as the HR-TEM images and elemental mapping of the nanosheets, the optical, cross-sectional, and surface SEM images, the elemental composition, and the water contact angles of the membranes. There are also supplementary data related to the ion permeation experiments, such as the influence of feed concentration, membrane thickness, ScCO_2_ treatment procedure, and crown ether doping on the ion flux and selectivity. (Figures S1–S17, Tables S1 and S2).

## Abbreviations

The following abbreviations are used in this manuscript:

Tp	1,3,5-Triformylphloroglucinol
EB	Ethidium bromide
COF	Covalent Organic Frameworks
ScCO_2_	Supercritical CO_2_
