# Supplementary material for: Regulating the Crystalline Structure and Ion Affinity of Covalent Organic Frameworks for Enhanced Lithium/Magnesium Separation
Source: Biomimetics (Basel). 2026 Mar 3;11(3):177. doi: 10.3390/biomimetics11030177 (PMC13024126; doi:10.3390/biomimetics11030177)
Supplement: Supplementary file 1 [file biomimetics-11-00177-s001.zip › Supporting Information.pdf]

Supporting Information

Regulating the Crystalline Structure and Ion Affinity of Covalent Organic Frameworks for Enhanced Lithium/Magnesium Separation

Chuncai Wang[1,2], Shiwen Bao[1,2], Yanfeng Gong[1,2], Lei Yu[1,2], Zizhe Xu[1], Chul. B Park[1], Kunyan Sui[1], Jun Gao[2,3,], and Xueli Liu[1,]**

[1]College of Materials Science and Engineering, Key Laboratory of Marine Bio-based Fibers of Shandong Province, Key Laboratory of Shandong Provincial Universities for Advanced Fibers and Composites, Qingdao University, Qingdao 266071, China

[2]Qingdao Institute of Bioenergy and Bioprocess Technology, Chinese Academy of Sciences, Qingdao 266101, China

[3] Shandong Energy Institute, Qingdao 266101, China

*Corresponding author

Email**:** jun.gao@qibebt.ac.cn (J. Gao); liuxl@qdu.edu.cn (X. Liu)

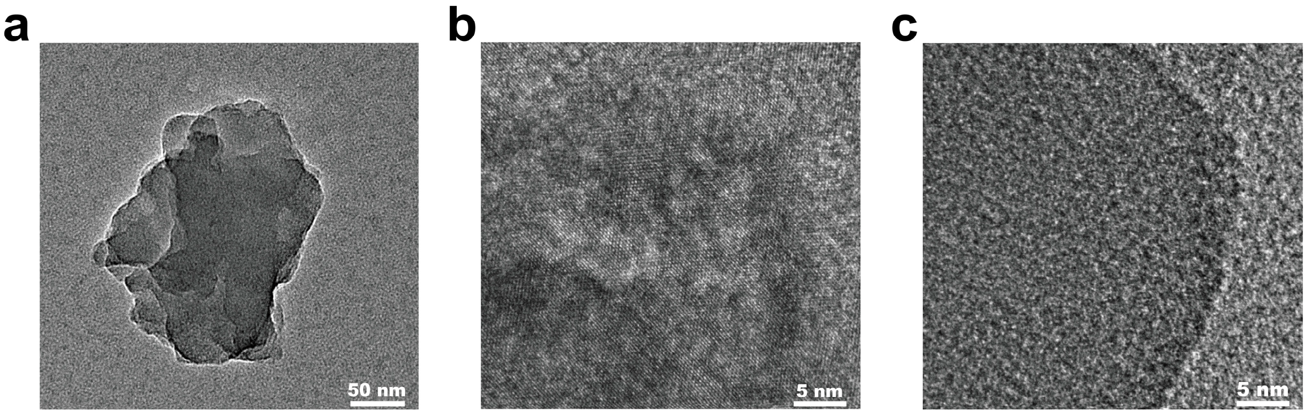

Figure S1: HR-TEM characterization of the TpEB COF nanosheets. (**a**) TEM image of the COF nanosheet. The nanosheets own both ordered region (**b**) and amorphous region (**c**).

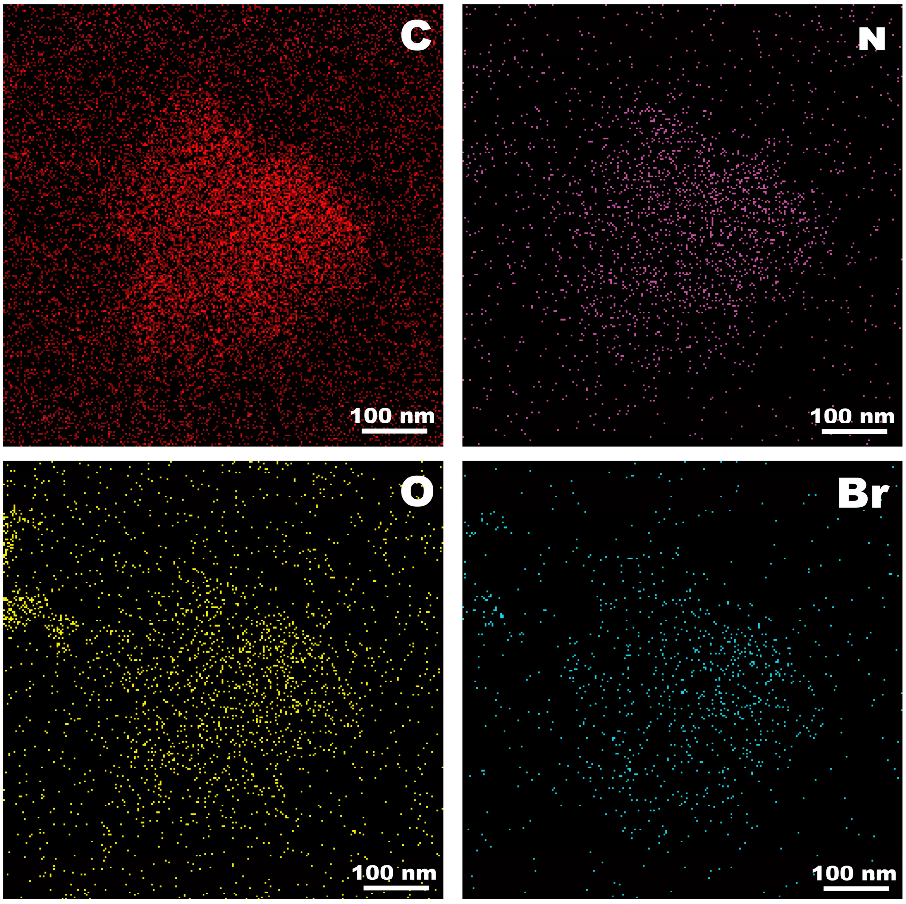

Figure S2: TEM elemental mapping of the TpEB COF nanosheets.

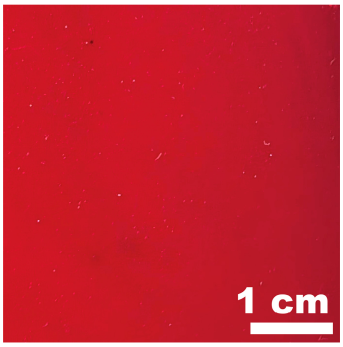

Figure S3: Optical image of the pristine TpEB COF membrane.

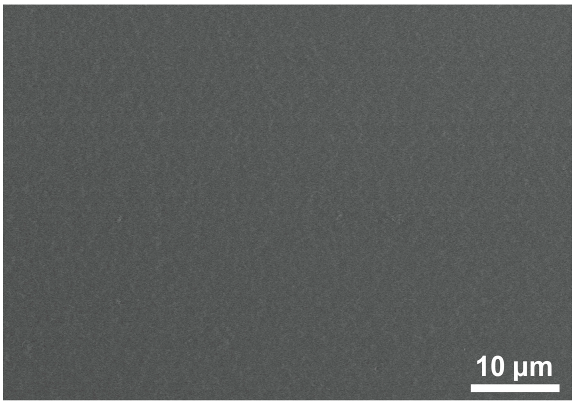

Figure S4: Surface SEM image of the pristine TpEB COF membrane.

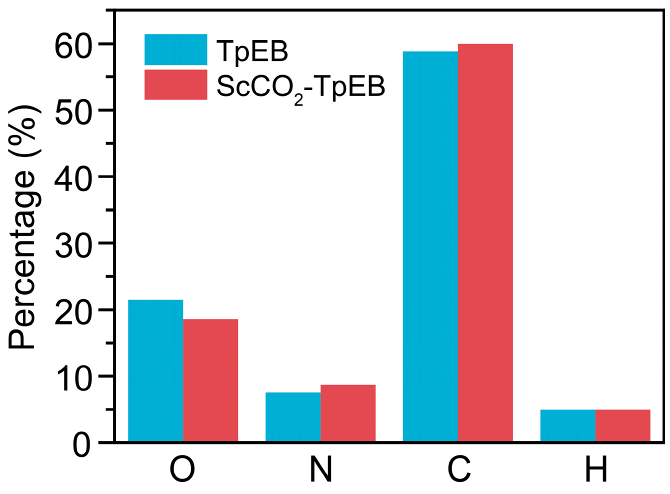

Figure S5: EDS analysis of the element composition of the pristine and the $ScCO_2$-TpEB COF membranes.

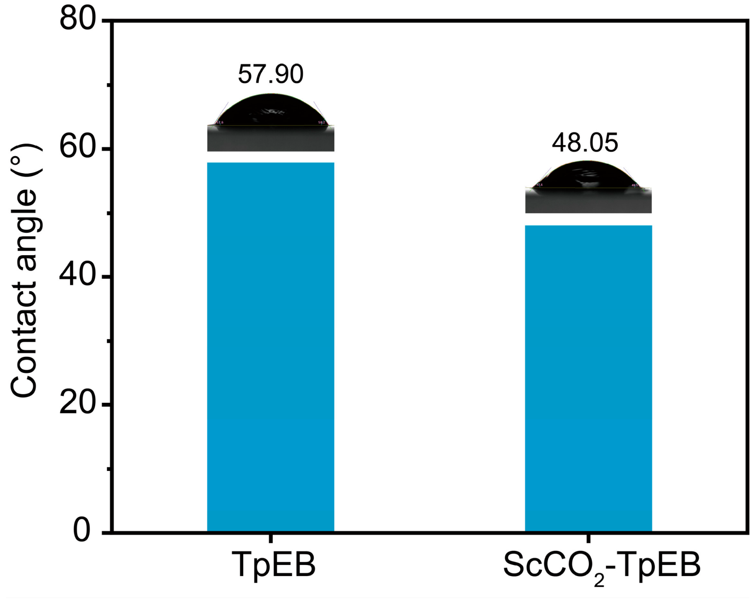

Figure S6: Contact angle measurements of the pristine and the $ScCO_2$-TpEB COF membranes.

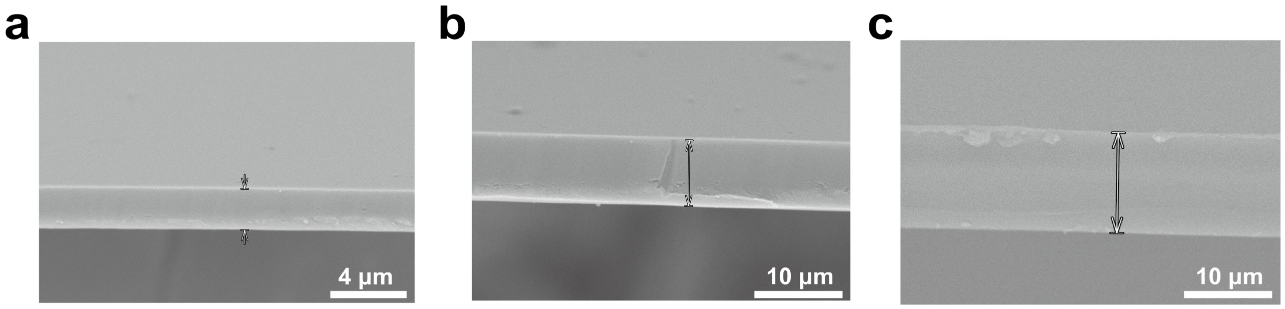

Figure S7: Cross-sectional SEM images of the ScCO$_2$-COF membranes with varying thickness. The thickness was (**a**) 2 μm, (**b**) 7 μm, and (**c**) 11 μm, respectively.

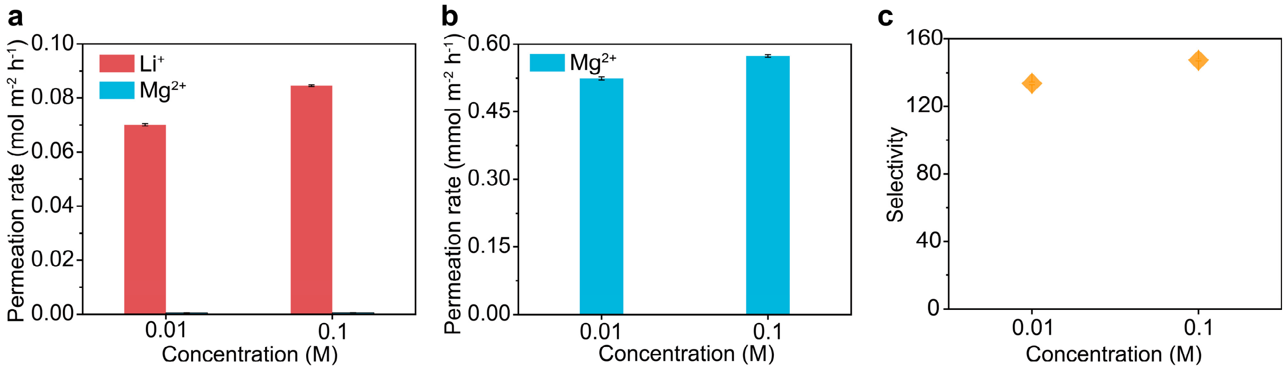

Figure S8: Influence of feed concentration on the selective Li^+ transport of the ScCO$_2$ COF membrane. (**a-b**) The influence of feed solution concentration on the Li^+ and Mg^{2+} permeation rates. (**b**) represents the results of Mg^{2+} in (a). (**c**) The influence of feed solution concentration on the Li^+/Mg^{2+} selectivity.

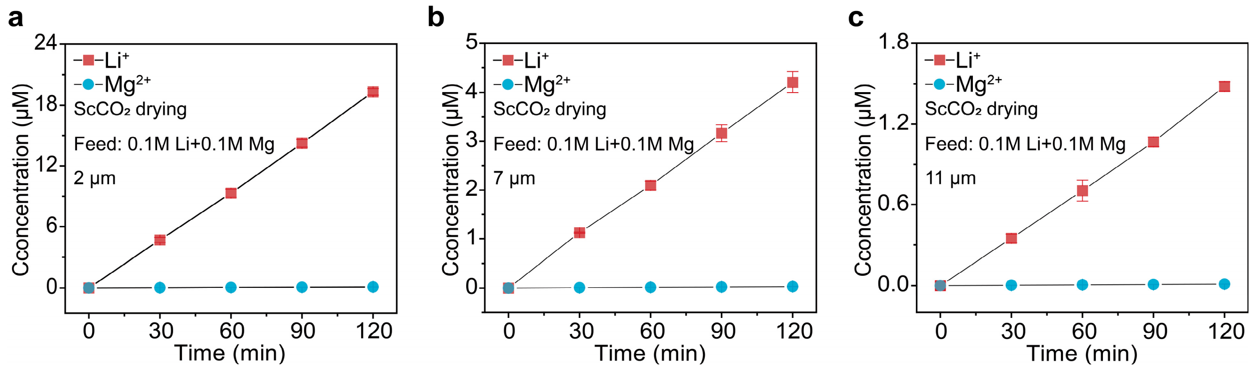

Figure S9: Influence of membrane thickness on the selective Li^+ transport of the $ScCO_2$-COF membranes. (a-c) The concentration of Li^+ and Mg^{2+} in the permeate as a function of time for the membrane with a thickness of (**a**) 2 μm, (**b**) 7 μm, and (**c**) 11 μm, respectively.

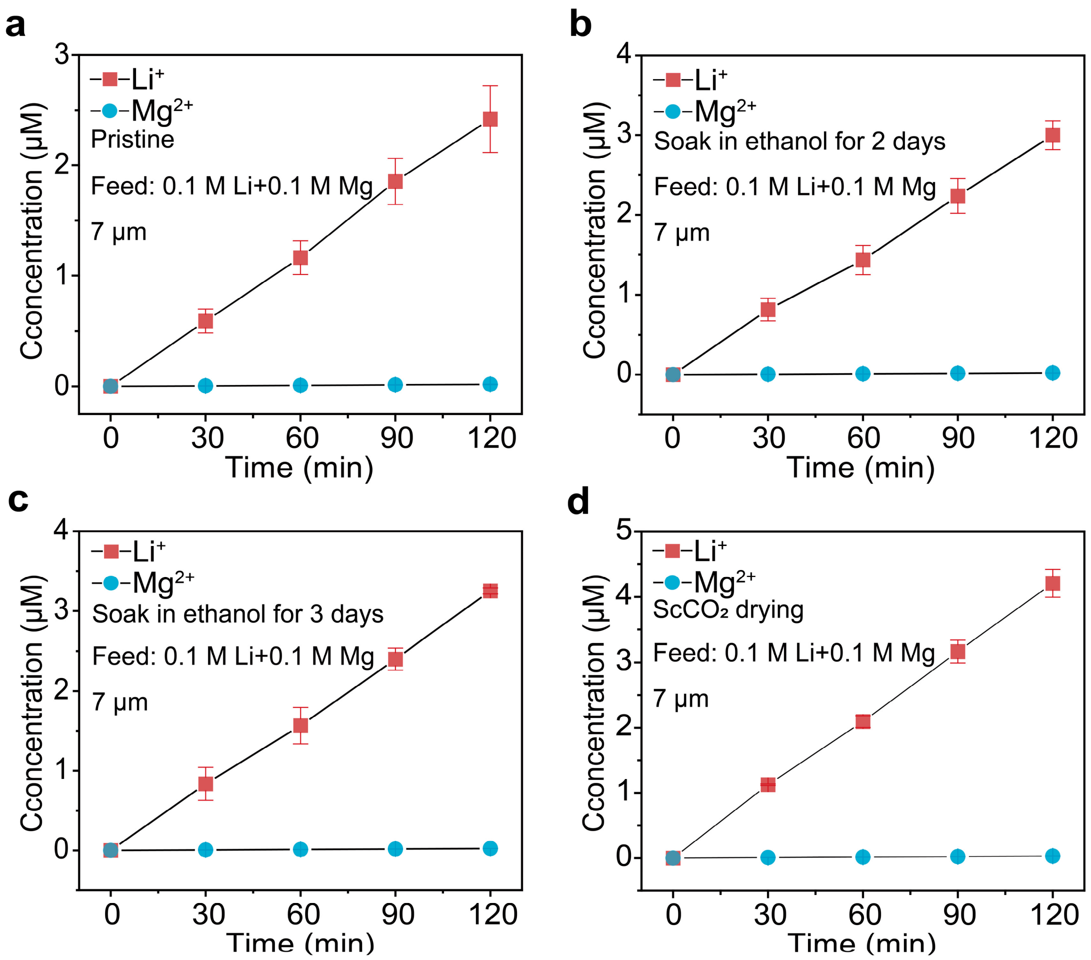

Figure S10: The influence of $ScCO_2$ drying on the selective Li^+ transport of the COF membrane with a 7 μm thickness. (**a-d**) Concentrations of Li^+ and Mg^{2+} in the permeate along with time, for the pristine TpEB COF membrane (a), the membrane soaked in ethanol for 2 (b) and 3 (c) days before $ScCO_2$ drying, and the membrane after $ScCO_2$ drying (d).

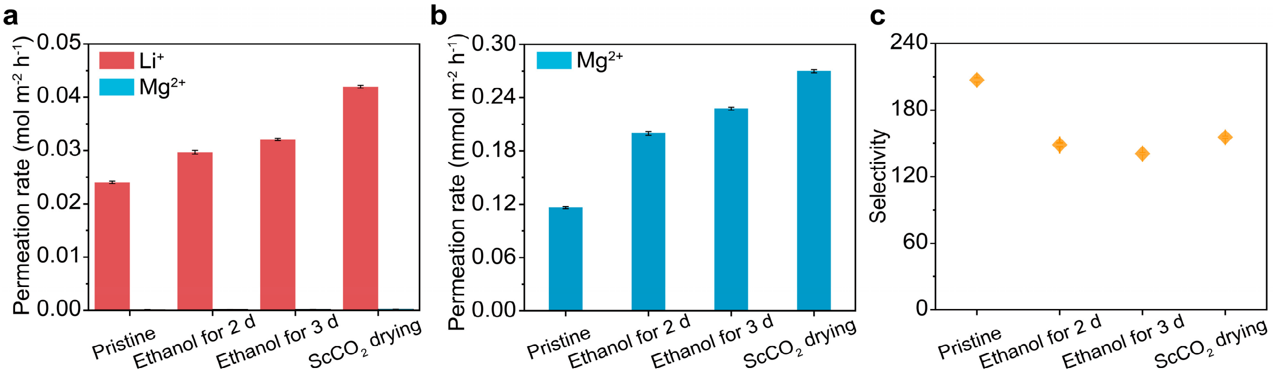

Figure S11: The influence of $ScCO_2$ drying on the selective Li^+ transport of the COF membrane with a 7 µm thickness (**a**) Comparison of the permeation rates of Li^+ and Mg^{2+}, for the pristine, ethanol-soaked, and $ScCO_2$ treated COF membranes. (**b**) The enlarge diagram, showing the permeation rates of Mg^{2+} corresponding to those in (a). (**c**) The corresponding Li^+/Mg^{2+} selectivity concluded from (a-b).

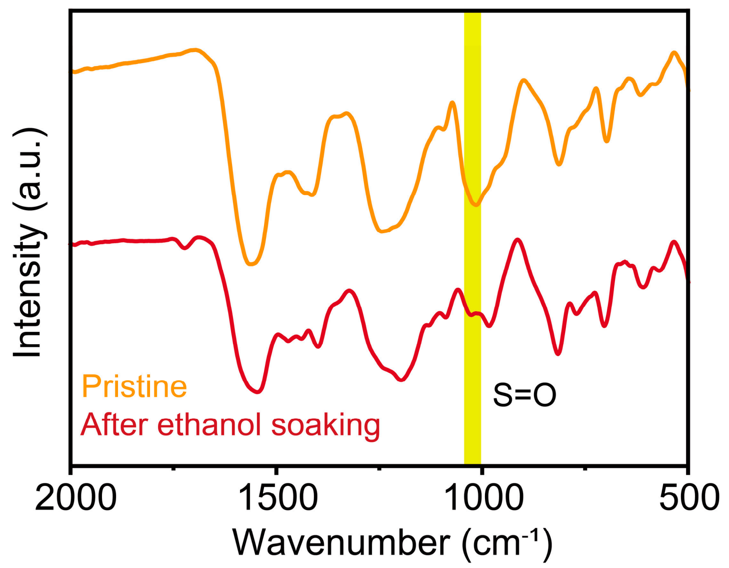

Figure S12: FT-IR spectra of the pristine TpEB COF membrane (yellow curve) and the TpEB COF membrane after ethanol soaking (red curve). The attenuation of the S=O stretching vibration at ~1025 cm^{-1} after ethanol soaking indicates the effective removal of residual DMSO from the membrane.

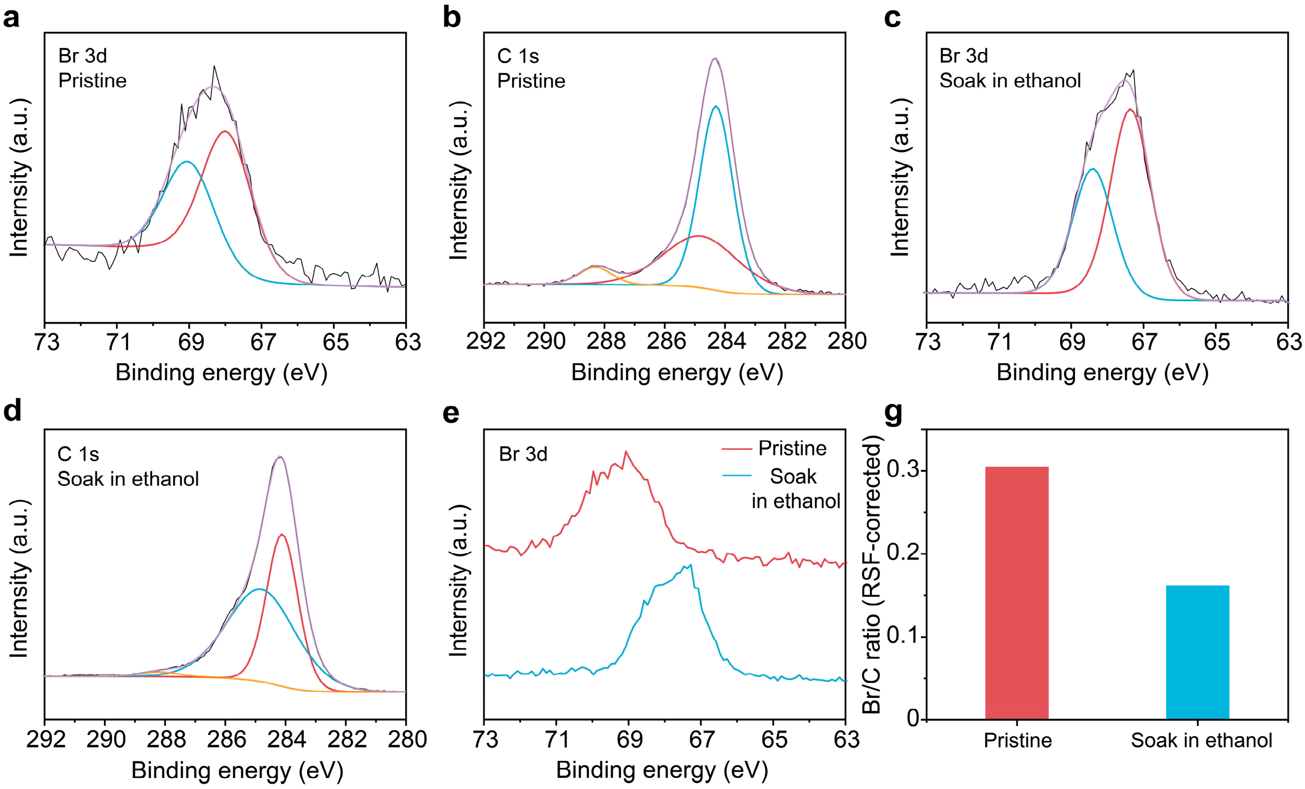

Figure S13: XPS analysis of TpEB COF membranes before and after ethanol soaking. (a) High-resolution Br 3d spectrum of the pristine membrane with curve fitting. (b) High-resolution C 1s spectrum of the pristine membrane with curve fitting. (c) High-resolution Br 3d spectrum of the ethanol-soaked membrane with curve fitting. (d) High-resolution C 1s spectrum of the ethanol-soaked membrane with curve fitting. (e) Comparison of Br 3d spectra for pristine and ethanol-soaked membranes (spectra vertically offset for clarity). (g) RSF-corrected Br/C ratios derived from background-subtracted integrated peak areas. The ethanol treatment results in a significant decrease in surface Br content.

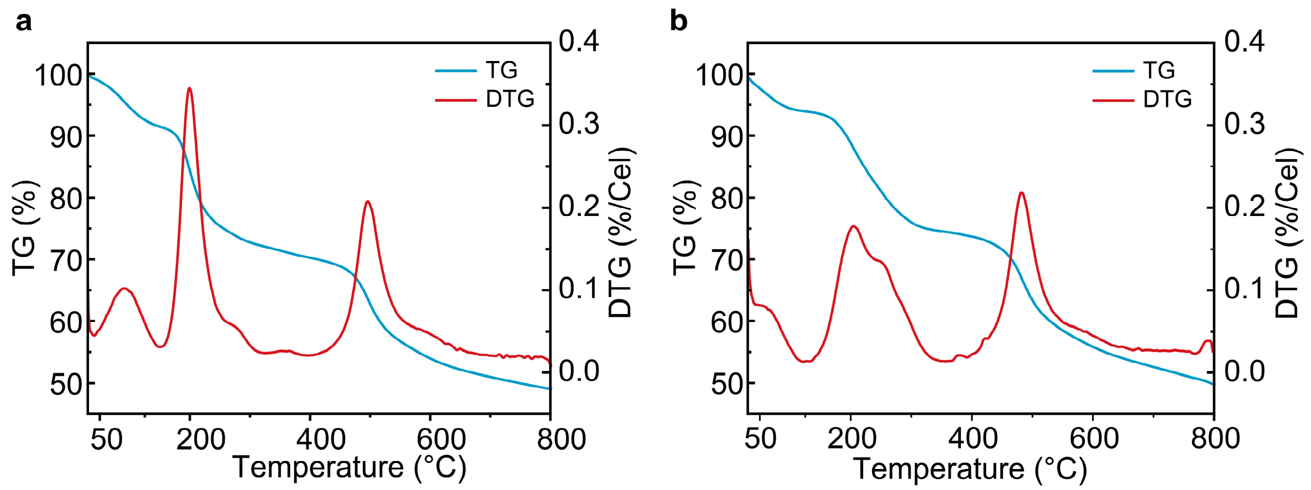

Figure S14: TGA analysis of the pristine TpEB COF membrane (a) and the TpEB COF membrane subjected to ethanol soaking followed by ScCO$_2$ drying (b). The markedly reduced weight loss in the low-temperature region (120–250 °C) after the treatment indicates the effective removal of residual DMSO and weakly bound monomers trapped within the membrane.

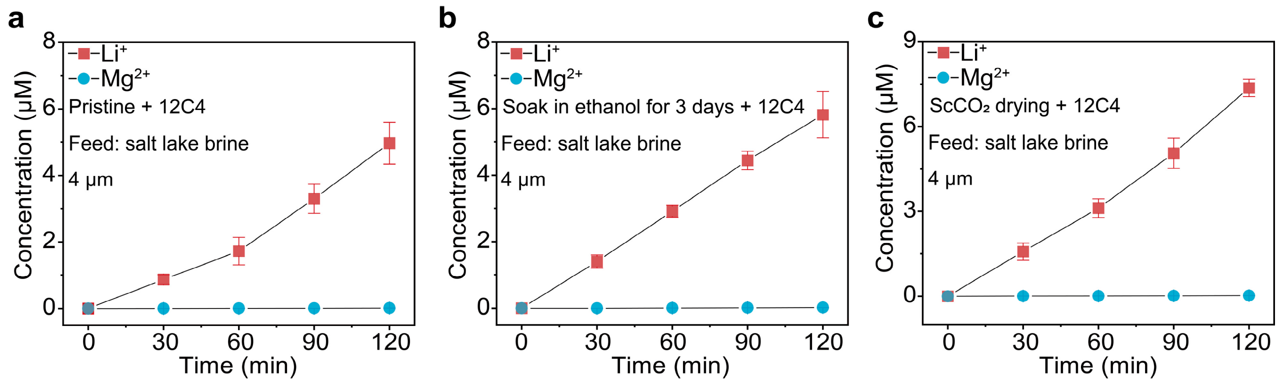

Figure S15: The influence of ScCO$_2$ drying on the selective Li^+ transport of the COF membrane doped with 12C4 crown ether. (**a-c**) Concentrations of Li^+ and Mg^{2+} in the permeate along with time, for the pristine membrane (a), the one soaked in ethanol for 3 (b) days before ScCO$_2$ drying, and the membrane after ScCO$_2$ drying (c).

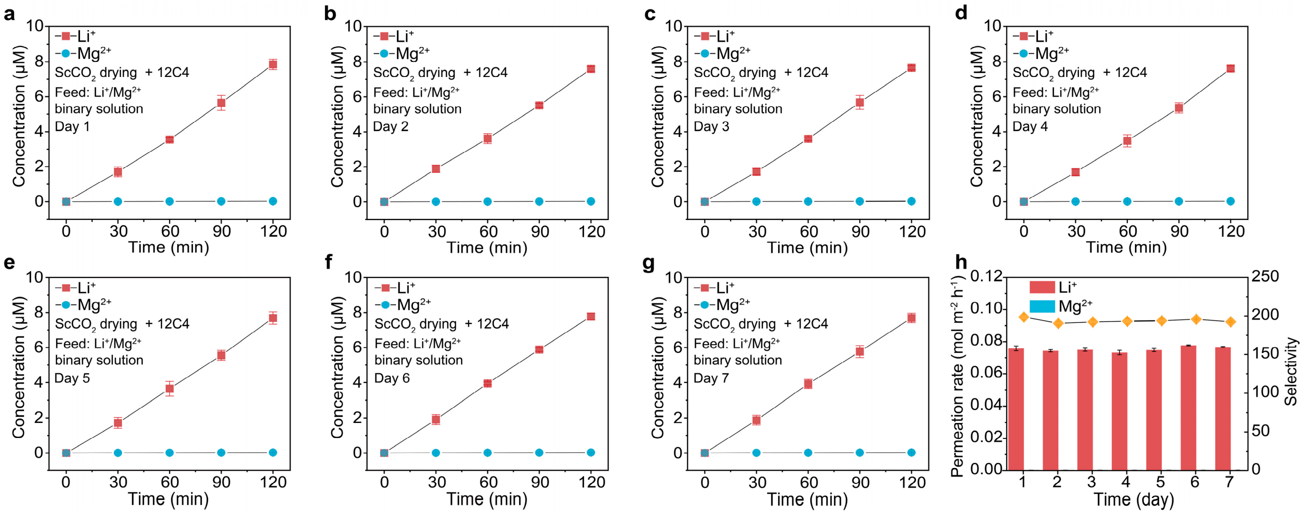

Figure S16: Long-term Li^+ separation performance of the crown ether-functionalized $ScCO_2$-COF membrane using Li^+/Mg^{2+} binary solution as the feed. (a-g) Concentrations of Li^+ and Mg^{2+} in the permeate as a function of time. (**h**) Comparison of the permeation rates of Li^+ and Mg^{2+} and the corresponding Li^+/Mg^{2+} selectivity.

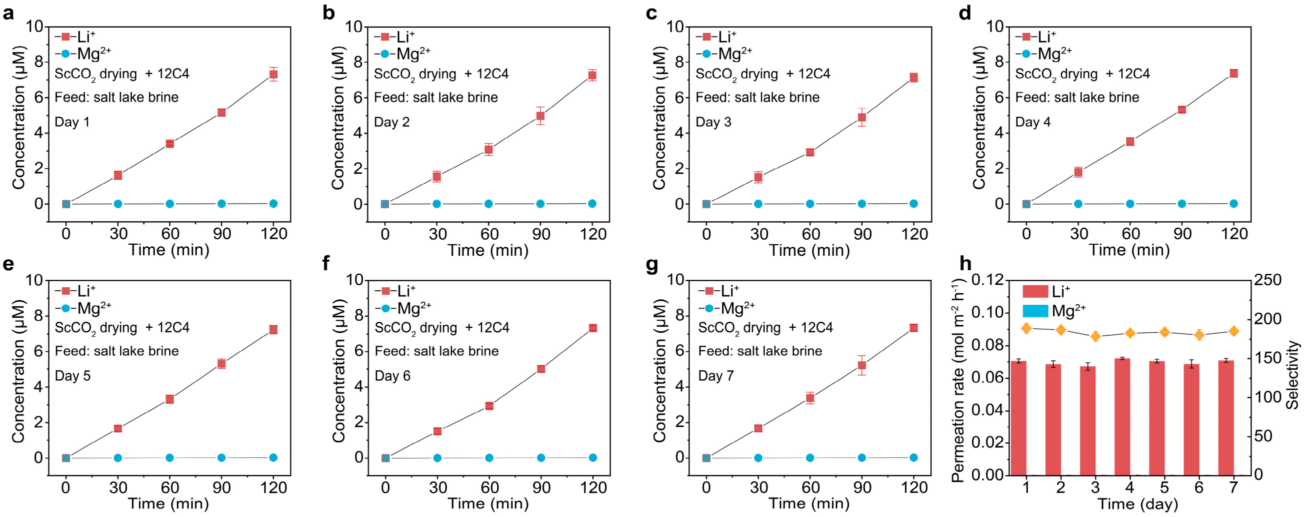

Figure S17: Long-term Li^+ separation performance of the crown ether-functionalized $ScCO_2$-COF membrane using real salt lake brine as the feed. (a-g) Concentrations of Li^+ and Mg^{2+} in the permeate as a function of time. (**h**) Comparison of the permeation rates of Li^+ and Mg^{2+} and the corresponding Li^+/Mg^{2+} selectivity.

Table S1: Ionic composition of the water from Qinghai Lake, China.

Ion type	Li^+	Na^+	K^+	Ca^{2+}	Mg^{2+}
Concentration (M)	0.035	2.17	0.38	0.2	0.022

Table S2: Comparison of Li^+/Mg^{2+} separation performance of TpEB-ScCO_2 and reported membranes under concentration gradient

Membrane Type	Feed Solution	Li^+ permeation rate (mol m^{-2} h^{-1})	Li^+/Mg^{2+} selectivity	Reference
TpEBr	0.2 M Li^++ 0.2 M Mg^{2+}	0.05	41.1	[1]
TpPa-SO_3H	0.1 M Li^++ 0.1 M Mg^{2+}	0.0025	1000	[2]
p-TpPa-C≡CH	0.05 M Li^++ 0.05 M Mg^{2+}	0.025	102.5	[3]
p-ETTBPa-C≡CH	0.05 M Li^++ 0.05 M Mg^{2+}	0.08	51.3	[3]
TpPa-SO_3H	0.1 M Li^++ 0.1 M Mg^{2+}	0.121	174	[4]
TG-1	0.1 M Li^++ 0.1 M Mg^{2+}	0.003402	423	[5]
TpBDMe$_2$	0.1 M Li^++ 0.1 M Mg^{2+}	0.0383	36	[6]
Cu-SAV	0.25 M Li^+/ 0.25 M Mg^{2+}	1.02	34	[7]
JGOM	0.2 M Li^++ 0.2 M Mg^{2+}	0.24	43.5	[8]
MMTM-LC	0.1 M Li^++ 0.1 M Mg^{2+}	0.11	38.13	[9]
MMTM-LC	Lop Nor brine	0.006	24.88	[9]
CMM	30 mM Li^++ 0.5 M Mg^{2+}	0.0059	62	[10]
TpEB-ScCO_2	0.1 M Li^++ 0.1 M Mg^{2+}	0.085	147	This work
TpEB-ScCO_2+12C4	Salt lake brine	0.081	187	This work

References

1. Wang, R.; Ding, L.; Xue, J.; Wu, H.; Cai, C.; Qiao, Z.; Caro, J.; Wang, H. Engineering of covalent organic framework nanosheet membranes for fast and efficient ion sieving: Charge-Induced Cation Confined Transport. *Small Methods* **2024**, *9*, doi:10.1002/smtd.202401111.
2. Wang, G.; Shao, L.; Zhang, S. Membrane-ion interactions creating dual-nanoconfined channels for superior mixed ion separations. *Adv. Mater.* **2025**, *37*, 2414898, doi:10.1002/adma.202414898.
3. Wang, M.; Wu, Z.H.; Zhou, G.Z.; Wu, X.Q.; Li, S.; Ma, T.Y.; Ren, Y.; Hai, G.; Wang, B.; Li, D.S. Partitioning COF membrane channels into ultramicroporous and π-Electron-Rich compartments for multicomponent ion separations. *Angew. Chem. Int. Ed.* **2025**, *64*, doi:10.1002/anie.202514179.
4. Hu, X.-F.; Jiang, T.; Fan, H.; Guan, Y.-F.; Chen, J.-J.; Yu, H.-Q.; Elimelech, M. Dual-regulated covalent organic framework membranes with near-theoretical pore sizes for angstrom-scale ion separations. *Sci. Adv.* **2025**, *11*, eady3587, doi:10.1126/sciadv.ady3587.
5. Liu, C.; Li, L.; Li, L.; He, X.; Matsuyama, H. Tuning the size and ion affinity of channels within guanidinium-based covalent organic framework membranes for efficient Li^+/Mg^{2+} separation. *J. Membr. Sci.* **2025**, *734*, doi:10.1016/j.memsci.2025.124433.
6. Sheng, F.; Wu, B.; Li, X.; Xu, T.; Shehzad, M.A.; Wang, X.; Ge, L.; Wang, H.; Xu, T. Efficient ion sieving in covalent organic framework membranes with Sub-2-Nanometer channels. *Adv. Mater.* **2021**, *33*, doi:10.1002/adma.202104404.
7. Zhang, L.; Huang, Z.; Chen, Y.; Li, F.; Li, G.; Zhang, F. Highly selective lithium-ion separation by regulating ion transport energy barriers of vermiculite membranes. *ACS Nano* **2025**, *20*, 1345-1356, doi:10.1021/acsnano.5c17718.
8. Wang, S.; Fang, C.; Huang, Y.; Yi, R.; Wu, M.; Wang, Y.; Li, F.; Zhu, L.; Liang, S.; Chen, L. Bio-Inspired 2D asymmetric nanochannels for high-resolution Li^+/Mg^{2+} separation. *Angew. Chem. Int. Ed.* **2025**, *64*, doi:10.1002/anie.202512310.
9. Jiang, X.; Zhang, L.; Miao, Y.; Chen, L.; Liu, J.; Zhang, T.; Cheng, S.; Song, Y.; Zhao, Y. Intrinsic roles of nanosheet characteristics in two-dimensional montmorillonite membranes for efficient Li^+/Mg^{2+} separation. *Water Res.* **2025**, *276*, doi:10.1016/j.watres.2025.123291.

10. Zhang, G.; Hu, G.; Li, J.; Feng, G.; Cui, Y.; Chen, Z.; Liu, Z.; Cui, Y. Design strategies for ion-sieving charge mosaic membranes toward sustainable lithium extraction. *Proc. Nat. Acad. Sci* **2025**, *122*, e2511666122, doi:10.1073/pnas.2511666122.
